# Attenuation of Methotrexate-Induced Embryotoxicity and Oxidative Stress by Ethyl Pyruvate 

**DOI:** 10.22074/ijfs.2016.4914

**Published:** 2016-06-01

**Authors:** Gholamreza Najafi, Elham Atashfaraz, Farah Farokhi

**Affiliations:** 1 Department of Basic Sciences (Anatomy and Embryology), Faculty of Veterinary Medicine, Urmia University, Urmia, Iran; 2 Department of Biology, Faculty of Science, Urmia University, Urmia, Iran

**Keywords:** Ethyl Pyruvate, In Vitro Fertilization, Methotrexate, Testis

## Abstract

**Background:**

Methotrexate (MTX), as an anti-folate agent, is widely used in the treatment of rheumatic disorders and malignant tumors, however it damages reproductive sys- tem in mice. The aim of this research was to study the effects of ethyl pyruvate (EP) on embryo development and oxidative stress changes in the testis of mice treated with MTX.

**Materials and Methods:**

In this experimental study, thirty-two adult male Naval Medical Research Institute mice, with average weight of 26 ± 2 g, were divided into four groups. The first group (control) received distilled water (0.1 ml/mice/day), while the second group was intraperitoneally (IP) treated with 20 mg/kg MTX once per week. The third group was IP treated with 40 mg/kg/day EP, and the fourth group was IP treated with both 20 mg/kg MTX and 40 mg/kg/day EP for 30 days. At the end of treatment fertilization rate and embryonic development were evaluated. Differences between these groups were assessed by ANOVA using the SPSS software package for Windows with a Tukey-Kramer multiple post-hoc comparison test.

**Results:**

MTX treatment caused significant (P<0.05) increase in malondialdehyde (MDA) and reduced catalase (CAT), as well as leading to *in vitro* fertilization (IVF) and embryonic development. The improved effects of EP on the IVF were determined by the reduced level of MDA (index of oxidative stress) and significant increased level of CAT (a key antioxidant). We observed significant increase in fertilization rate and embryonic development in the treated group with both MTX and EP.

**Conclusion:**

It is suggested that EP can be useful in ameliorating testicular damages and embryotoxicity induced by MTX. These effects could be attributed to its antioxidant properties.

## Introduction

Infertility has been one of the most controversial medical and social issues. It was previously proposed as a punishment in some civilizations, while it is an illness (1). In addition, lack of the knowledge on medicine is generally the main factor to always consider female as responsible for reproductive system failure. Several investigations within the last decade help scientists and clinicians distinguish crucial parameters involved in couple’s infertility, one of which is sperm malfunction in male (2). About 5-15% of couples are infertile, out of whom about 50% of infertility is caused by male disorders (3). Methotrexate (MTX) is an anti-neoplastic agent which is used alone or in combination with other agents to treat severe psoriasis and rheumatoid arthritis (4). It is also used against a broad range of neoplastic disorders including acute lymphoblastic leukemia, non-Hodgkin’s lymphoma, breast cancer and testicular tumors (5,6). 

It is well demonstrated that cancer chemotherapeutic drugs could cause acute toxic side-effects in multiple organs such as gastrointestinal tract, lung, kidney, liver, testes and skin. Several currently available anti-cancer drugs, specifically MTX, generally function by destruction of particular proliferating cells with both normal and malignant origins (7,8). MTX might also cause primary infertility by affecting hypothalamic-pituitary-gonadal axis or gonads, directly (9). This drug is bound to dihydrofolate-reductase enzyme, which inhibits DNA replication and prevents the synthesis of purines, thymidilate as well as glycine, eventually leading to cell death (10). It has been manifested that MTX, as a cytotoxic agent, should be considered a potential occupational reproductive hazard which is harmful for the fetus, and could potentially cause human carcinogenesis (11). Study of this drug in animal models showed cytotoxicity, altered spermatogenesis, degeneration of spermatocytes in sertoli and leydig cells (12,13). 

On the other hand, pyruvate is a key intermediate metabolite of glucose, playing role as a potent anti-oxidant and free scavenger. It has been demonstrated that pyruvate has also an anti-inflammatory effect, both *in vitro* and in vivo (14). While being a good scavenger for hydrogen peroxide and superoxide radicals (15), the efficiency of pyruvate as a therapeutic agent is limited by its instability (16). In comparison, ethyl pyruvate (EP), a simple aliphatic ester derived from pyruvic acid, is safer and more stable than pyruvate (17). Similar to pyruvate, EP can rapidly and stoichiometrically scavenge hydrogen peroxide. Investigations showed that treatment with EP reduces oxidative stress both *in vitro* and in vivo models of ischemia/reperfusion injury (15). 

*In vitro*fertilization (IVF), is a safe and effective way and the last option for the infertile couples who have tried and failed to conceive using standard treatments such as surgery, fertility drugs and artificial insemination (18). The aim of this research was to study the effect of EP on embryo development and oxidative stress changes in mice testis treated with MTX. 

## Materials and Methods

### Animals

This was an experimental study on thirty two adult male Naval Medical Research Institute mice with 8-10 weeks of age and 26 ± 2 g average weight. The mice were purchased from animal house of Science Faculty of Urmia University (Urmia, Iran). All animals were kept under standard environmental conditions, including 22 ± 2°C room temperature, relative humidity of 50 ± 10% and a dark/light photoperiod of 12/12 hours, respectively. The animals had open access to standard diet pellets, water and libitum. This study was performed according to Ethical Committee Guidance for Research at Laboratory Animals of Urmia University. 

### Experimental design

Animals were randomly divided into four equal number groups and treated for 30 days. Group 1 (control) received 0.1 ml/mice/day distilled water intraperitoneally (IP). Group 2 mice were IP administered once per week with 20 mg/kg MTX. Group 3 was IP treated with 40 mg/kg/day EP. Group 4 received both MTX and EP treatment, with similar dosages to the individual treatments. 

### Oocyte collection

To perform assisted reproductive technique (ART) procedure, the oviduct ampullas was initially collected. Briefly, each male mouse (total number: 32) was mated with three female mice (total number: 96). Superovulation was induced in 6-7 weeks old female mice by IP injection of 10 IU pregnant mare serum gonadotropin (PMSG, Boxmeer, Netherlands), in addition to IP injection of 10 IU human chorionic gonadotropin (hCG, Folligon, Netherlands) after 48 hours. 12-14 hours after hCG injection, female mice were sacrificed by cervical vertebrae dislocation. The oviduct ampullas were removed and transferred to a petridish containing human tubular fluid (HTF, Sigma, St. Louis, USA) medium+4 mg/ml bovine serum albumin (BSA, Sigma, St. Louis, USA). The ampulla portions were distinguished by stereo microscope (Model TL2, Olympus Co., Japan), and oocytes were dissected out. 

### Preparation of culture media for in vitro fertilization

Required fertilization media was prepared and incubated for 12 hours in a gas mixture of 5% CO_2_ at 37°C, one day before conception. For each group, separate dishes were considered. Conception dishes with HTF medium were combined with 4 mg/ml BSA. Droplets of fertilization medium and washing HTF medium was prepared for the IVF dishes, followed by covering them with mineral oil (Sigma) and incubation at 37°C) for overnight. 

### Sperm preparation and insemination

24 hours after the last treatment, male mice were sacrificed by cervical dislocation. Using a ventral midline skin incision the epididymides were separated from the testicles. Afterward, caudal epididymis (cut into 2-3 pieces) was placed in 1 ml HTF medium with a combination of 4 mg/ml BSA and incubated at 37˚C for 60 minutes in a 5% CO_2_ incubator. Obtained sper-matozoa were capacitated by incubation at 37˚C and 5% CO_2_ for 1 hour. 

In addition, 12-14 hours after injection of HCG, female mice were sacrificed by cervical dislocation. The ampules of fallopian tubes were removed, put in the HTF medium with 37°C temperature. The oocytes were subsequently removed with dissecting techniques, followed by washing with the HTF and transferring to the fertilization droplets under mineral oil containing HTF and 4 mg/ml BSA. As soon as overtaking capacitation step of the sperms, they were added to the medium, in a concentration of 1×10^6^sperms/ml culture medium. 

### Assessment of fertilization and embryonic development

Using an inverted microscope (Model TL2, Olympus Co., Japan), fertilized oocytes were evaluated by appearance of the pronuclei (female and male) under magnitude of ×200. After denuding of granulosa cells and washing with HTF medium (100 µl), zygotes were transferred into pre-equilibrated fresh medium and cultured for five days. After 24 hours of zygote culture, the rate of two-cell embryos was assessed. Embryonic development was evaluated by inverted microscope after 120 hours culture *in vitro*. Intact, fragmented and/or lysed embryos, which have not been developed, were considered as "arrested embryos". 

### Catalase assay

Catalase (CAT) activity, based on the ability to decompose H_2_O_2_ in homogenized testicular tissue, was determined by Aebi method (19). By reducing the wavelength absorption at 240 nm in the absorption spectrum, decomposition of H_2_O_2_ was detectable. For this purpose, 30 mM hydrogen peroxide was used as substrate, followed by 50 mM phosphate buffer (pH=7) as an alternative substrate in the blank solution. The assay solution contained 2 ml tissue homogenate and 1 ml hydrogen peroxide. The reaction was started by adding H_2_O_2_ and using a spectrophotometer (pharmaciaп novaspec, and biochrom, England) at a wavelength of 240 nm. Decrease in absorbance was evaluated for 30 seconds and the values were expressed in terms of (µg)/g testis tissue. 

### Lipid peroxidation assay (Malondialdehyde)

Malondialdehyde (MDA) is a product of lipid peroxidation. This experiment is widely used as an oxidative stress index. 300 µl of 10% trichloroacetic acid was added to 150 µl of the sample and centrifuged at 1000 rpm for 10 minutes at 4°C. 300 µl of the supernatant was subsequently transferred to a test tube containing 300 µl of 67% thiobarbituric acid. The mixture was incubated for 25 minutes at 100°C. After cooling the solution for 5 minutes, a pink color was appeared due to the reaction between MDA and TBA and absorbance was measured using a spectrophotometer at 535 nm wavelength (20). 

### Statistical analysis

SPSS-21 software was used for statistical analysis. The results were compared by one-way ANOVA, supplemented with a Tukey-Kramer multiple comparison test. Values of less than 0.05 were considered statistically significant. All results were expressed as means ± SEM. 

## Results

### Fertilization and embryonic development

**Fig.1:**
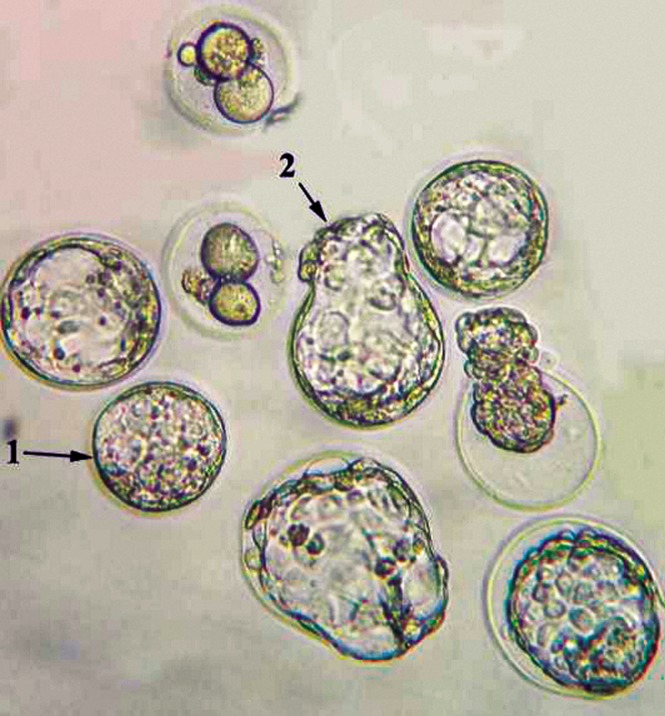
Representative image of blastocysts embryo (arrow number 1) and hatching embryo (arrow number 2) in the control group. The image was captured with ×200 microscope magnitude.

**Fig.2:**
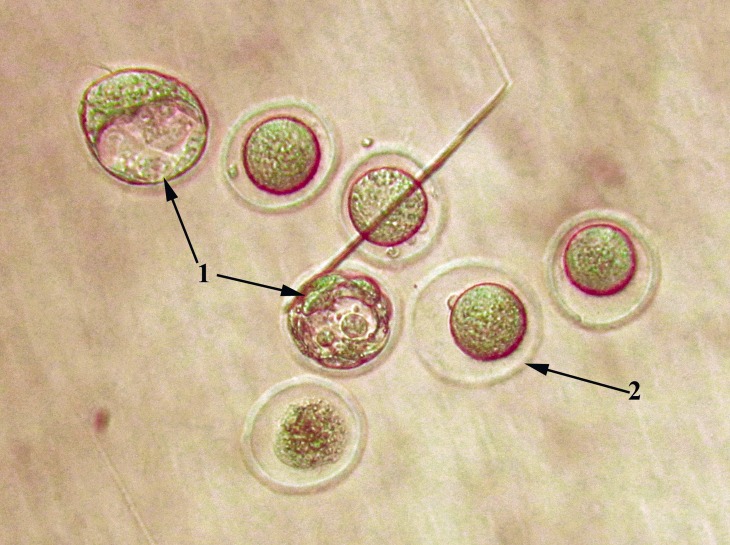
Representative image of blastocysts embryo (arrow number 1) and unfertilized oocytes (arrow number 2) in the Methotrexate (MTX) treated group. The image was captured with ×200 microscope magnitude.

**Fig.3:**
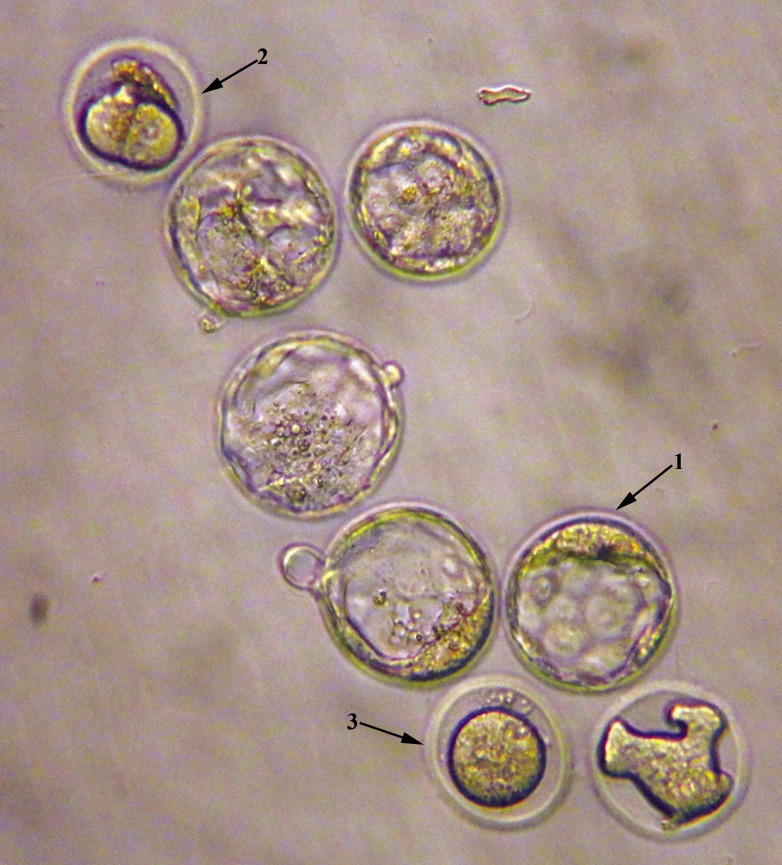
Representative image of blastocysts embryo (arrow number 1) and two-cell arrested embryo (arrow number 2) and unfertilized oocyte (arrow number 3) in the Methotrexate+ethyl pyruvate (MTX+EP) treated group. The image was captured with ×200 microscope magnitude.

In this study, after 30 days, treated mice group with 20 mg/kg of MTX showed significantly reduced rate of fertilization as well as percentage of blastocysts (Table 1,Fig.1). Compared to this group, there was a significant increase in fertilization rate and blastocysts percentage of mice treated with both MTX and EP (Table 1,Fig.2). Findings also demonstrated that the range of two-cell embryos was reduced in zygotes obtained from the MTX treated mice, although this change was not significant (Table 1,Fig.1). In addition, no significant difference was observed in two-cell embryos among the all groups. Furthermore, percentage of the arrested embryos was enhanced after MTX treatment compared to the control group, after 30 days treatment (P<0.05). Analysis of the mice treated with EP showed a significant decrease in the percentage of arrested embryos in comparison with MTX group (Table 1,Fig.3). 

**Table 1:** Effect of MTX and EP on fertilization and embryonic development of the mice


Parameters	Groups			
	Control	MTX	EP	MTX+EP

Number of oocytes	52	67	39	32
Fertilized oocytes (%)	92.11 ± 0.70	66.68 ± 2.29^a^	89.28 ± 1.26^b^	75.99 ± 2.70^a,b^
2-cell embryos (%)	83.93 ± 1.64	74.28 ± 2.47	81.90 ± 2.38	83.28 ± 3.67
Blastocysts (%)	82.46 ± 2.24	36.66 ± 7.69^a^	70.13 ± 4.75^b^	67.04 ± 7.48^b^
Arrested embryos (%)	9.65 ± 1.54	30.02 ± 5.40^a^	15.81 ± 1.77^b^	6.21 ± 1.18^b^


All the values are expressed as mean ± SEM (n=8). ^a^; Significant differences (P<0.05) compared to control group, ^b^; Significant differences (P<0.05) compared to MTX group, MTX; Metho- trexate, and EP; Ethyl pyruvate.

**Table 2:** Effect of MTX and EP on CAT and MDA levels in the adult male mice.


Parameters	Groups			
	Control	MTX	EP	MTX+EP

CAT (µ/g tissue)	0.54 ± 0.02	0.22 ± 0.01^a^	0.53 ± 0.02^b^	0.45 ± 0.02^a, b^
MDA (μmol/g tissue)	165.66 ± 4.63	357.33 ± 4.09^a^	187.00 ± 9.53^a b,^	203.66 ± 20.4^a,b^


All the values are expressed as Mean±SEM (n=8). ^a^; Significant differences (P<0.05) compared to control group, ^b^; Significant differences (P<0.05) compared to MTX group, MTX; Methotrexate, EP; Ethyl pyruvate, CAT; Catalase, and MDA; Malondialdehyde.

### Biochemical analysis

MTX administration for 30 days led to significant decrease in CAT level and increase in the level of MDA compared to the control. By the end of treatment period, EP administered groups showed significant increase in CAT and decrease in the MDA level compared to the MTX (Table 2). 

## Discussion

MTX, as a well-known anti-cancer agent with a narrow therapeutic window, is used for the treatment of malignant and non-malignant conditions (21). Although the cytotoxic effects of this agent has been determined in various organs (22). Several investigations implicated the potential negative effects of MTX on gonads. Thus, it is currently well known that administration of MTX can lead to reproductive system damages, including decreased epididymal and testicular weights, and reduced epididymal sperm counts and fertility rate (21). Sperm counts are a crude measure of fertility (23,24) and decrease in the count of sperm could often result from drug interference in the process of spermatogenesis and omission of sperm cells during different developmental stages (25). This agent also inhibits the synthesis of thymidylate, serine, and methionine, leading to disruption of DNA, RNA as well as protein function and consequently cell death (26). The sperm chromatin structure assay (SCSA) is an independent predictor of successful pregnancy (27). According to the results obtained from SCSA, Atashfaraz et al. (28) recently showed that MTX caused an increase in DNA fragmentation. 

A single exposure study, using IP route, indicated the transmissibility potential of MTX, substantiating its teratogenicity and embryo-lethality effects (29,30). The toxic effect of MTX on gonads was clearly determined by study on the histological evaluation of testes. Several symptoms, including increased disorganization, vacuolization, decreased spermatogonial and spermatid counts in the seminiferous tubules, indicate that MTX interferes in the process of spermatogenesis (21). In addition, similar studies have been conducted, reporting damages in spermatogonia as well as spermatocytes after repeated treatments with MTX in rats (8,31). In terms of male fertility, further to the counts, motility of sperms is also an important factor. Significant increase in lipid peroxidation along with sperm motility reduction might be a consequence of oxidative stress status observed by MTX administration (32,33). It has also been demonstrated that MTX can reduce the fertilization rate, percentage of blastocysts while increasing percentage of arrested embryos, probably due to the effect of this agent on oxidative stress induction. Recent medical advances have indicated that oxygen radicals and hydrogen peroxides are in association with the undesired adverse effects of several anti-tumor drugs (34). 

Free radicals are proposed to play a crucial role in MTX induced toxicity. In present study, we determined that level of MDA in the MTX-treated mice was significantly higher than the control group. This finding was consistent with several reports implicating that MTX induces oxidative stress in tissues by increasing MDA levels (35,34). The product of lipid peroxidation, MDA, and its level is widely used as an index of oxidative stress (36). Recent evidence has implicated oxidative stress as an etiological factor in the development of male infertility (37,38). Generally, men with significantly higher seminal reactive oxygen species (ROS) levels and lower antioxidant potential are diagnosed with idiopathic infertility (39). Zorn et al. (40) found that high seminal plasma ROS levels contribute to impaired sperm fertilization ability and lower pregnancy rates after IVF. 

CAT is an hemoprotein, catalyzing H_2_O_2_ reduction and protects tissues from ROS and hydroxyl radical levels growth (41). CAT, acting as a preventative antioxidant, plays an important role in protection against the deleterious effects of lipid peroxidation (42). In this study, we showed that MTX-treatment significantly reduced CAT level in testicular tissue. On the other hand, EP induced a reduction in MDA value and significantly increased CAT level in the mice treated with both MTX and EP. Given the close similarity of EP to an endogenous metabolite, the safety profile in animals and common application of this agent as a food supplement (43), it is unlikely to be harmful to humans. EP, a marker for oxidative stress both *in vitro* (44) and in vivo (45), inhibits lipid peroxidation. Tsung et al. (46) has recently indicated that it also decreases lipid peroxidation in liver tissue. Moreover, EP can react with ROS via both oxidative carboxylation and formation of hydroxylated adducts at the 3-carbon (47). 

Ultimately, we found that EP increased several fertility-related parameters, including fertilization rate and blastocysts percentage, and also reduced percentage of the arrested embryos probably due to its effect on the total antioxidant capacity and H_2_O_2_ scavengers. 

## Conclusion

The present study gives evidences of MTX-induced fertility damage and shows the capability of EP in preventing this damage by inhibiting the lipid peroxidation and improving the activity of antioxidant enzymes. The results of this study evaluated the efficacy of EP as a protective agent against the side-effects of chemotherapeutic agents. 
